# Effects of Radiation on Spinal Dura Mater and Surrounding Tissue in Mice

**DOI:** 10.1371/journal.pone.0133806

**Published:** 2015-07-27

**Authors:** Noriaki Yokogawa, Hideki Murakami, Satoru Demura, Satoshi Kato, Katsuhito Yoshioka, Miyuki Yamamoto, Shoichi Iseki, Hiroyuki Tsuchiya

**Affiliations:** 1 Department of Orthopaedic Surgery, Graduate School of Medical Science, Kanazawa University, 13–1 Takara-machi, Kanazawa, 920–8641, Japan; 2 Department of Histology and Embryology, Graduate School of Medical Science, Kanazawa University, 13–1 Takara-machi, Kanazawa, 920–8641, Japan; University of Louisville, UNITED STATES

## Abstract

**Purpose:**

Spinal surgery in a previously irradiated field carries increased risk of perioperative complications, such as delayed wound healing or wound infection. In addition, adhesion around the dura mater is often observed clinically. Therefore, similar to radiation-induced fibrosis—a major late-stage radiation injury in other tissue—epidural fibrosis is anticipated to occur after spinal radiation. In this study, we performed histopathologic assessment of postirradiation changes in the spinal dura mater and peridural tissue in mice.

**Materials and Methods:**

The thoracolumbar transition of ddY mice was irradiated with a single dose of 10 or 20 Gy. After resection of the irradiated spine, occurrence of epidural fibrosis and expression of transforming growth factor beta 1 in the spinal dura mater were evaluated. In addition, microstructures in the spinal dura mater and peridural tissue were assessed using an electron microscope.

**Results:**

In the 20-Gy irradiated mice, epidural fibrosis first occurred around 12 weeks postirradiation, and was observed in all cases from 16 weeks postirradiation. In contrast, epidural fibrosis was not observed in the nonirradiated mice. Compared with the nonirradiated mice, the 10- and 20-Gy irradiated mice had significantly more overexpression of transforming growth factor beta 1 at 1 week postirradiation and in the late stages after irradiation. In microstructural assessment, the arachnoid barrier cell layer was thinned at 12 and 24 weeks postirradiation compared with that in the nonirradiated mice.

**Conclusion:**

In mice, spinal epidural fibrosis develops in the late stages after high-dose irradiation, and overexpression of transforming growth factor beta 1 occurs in a manner similar to that seen in radiation-induced fibrosis in other tissue. Additionally, thinning of the arachnoid barrier cell layer was observed in the late stages after irradiation. Thus, consideration should be given to the possibility that these phenomena can occur as radiation-induced injuries of the spine.

## Introduction

Radiotherapy has been widely used for spinal metastases [1, 2], and is often performed as palliative treatment to improve patient quality of life by alleviating pain, mitigating nerve compression symptoms, and decreasing the likelihood of pathological bone fracture through local tumor control [3–5]. Until recently, presence of spinal metastases was regarded as an end-stage state; however, recent advances in multidisciplinary treatments for various cancers have led to an increase in the number of cases with a longer-term prognosis. Therefore, full consideration must be given to adverse events associated with radiotherapy, especially late-stage radiation injury in long-term survivors. Currently, effective treatments for late-stage radiation injury are lacking, and such injuries may develop into life-threatening complications [6, 7].

Recurrence of pain and nerve compression due to local tumor relapse after radiotherapy is a problem for long-term survivors of spinal metastases. Surgical treatment is often selected in these cases because repeat irradiation bears the risk of radiation myelopathy [8, 9]. However, late-stage radiation injury is associated with perioperative complications, such as delayed wound healing or wound infection [10, 11]. In addition, research at our facility has indicated that dural injury and postoperative cerebrospinal fluid (CSF) leakage, probably due to adhesion around the dura mater, are frequently observed during surgery for spinal tumors after radiotherapy [12]. Therefore, similar to radiation-induced fibrosis—a major late-stage radiation injury in other tissue [13–15]—epidural fibrosis is anticipated to precede dural injury in the spine; however, this has not yet been investigated. In this study, we performed histopathologic assessment of temporal changes in the spinal dura mater and peridural tissue following irradiation in mice, with a focus on epidural fibrosis.

## Materials and Methods

### Study design

**Fig 1:**
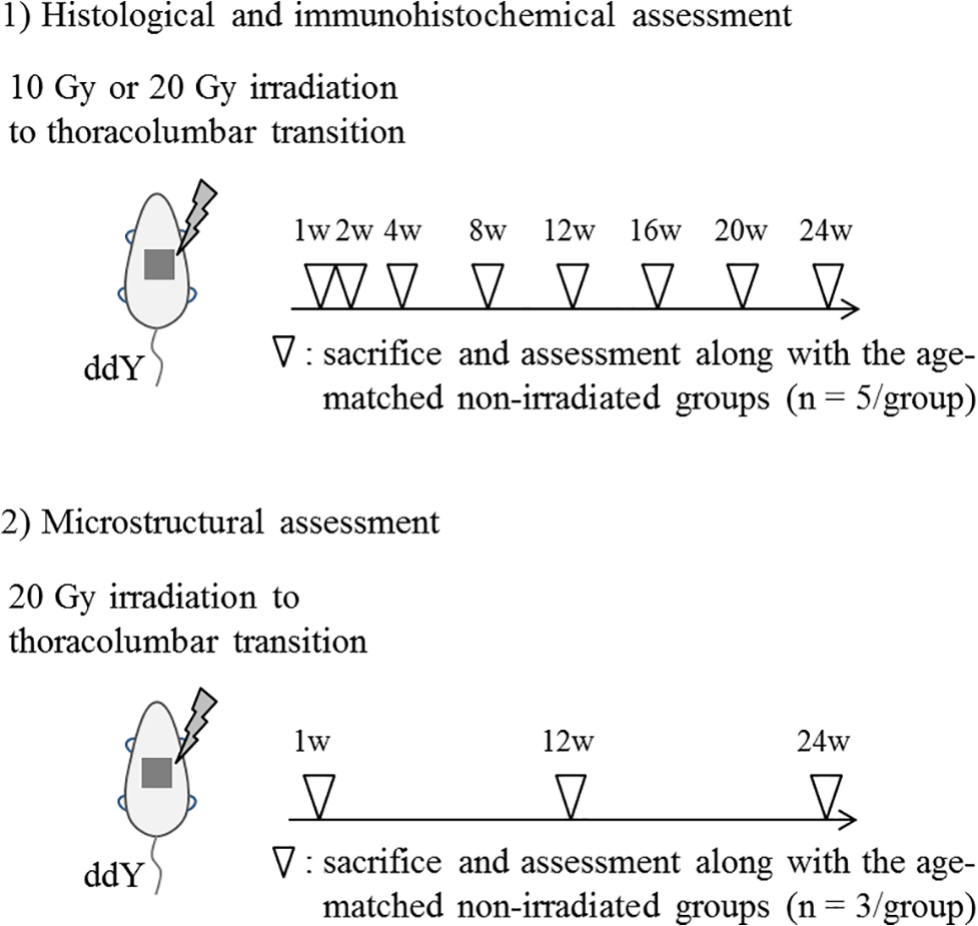
Experimental protocol.

This study was conducted with approval from the Committee of Animal Care and Experimentation at Kanazawa University (Kanazawa, Japan, AP-122282). All surgeries were performed under sodium pentobarbital anesthesia, and all efforts were made to minimize suffering. Fig 1 shows the experimental protocol. Ten-week-old ddY mice (body mass, 30–32 g) purchased from Japan SLC (Shizuoka, Japan) were randomly allocated into irradiated and nonirradiated groups. The irradiated groups received a single external irradiation dose of 10 or 20 Gy at 150 kV and 20 mA to the thoracolumbar transition using an X-ray irradiation device for small animals (HITACHI MBR-1520R-3, Tokyo, Japan). X-ray irradiation was beamed through 0.5-mm aluminum and 0.5-mm copper filters. After general anesthesia by intraperitoneal administration of pentobarbital (50 mg/kg), the mice were immobilized in the lateral decubitus position and then irradiated under a lead plate containing 20 × 20-mm openings to irradiate the thoracolumbar transition alone. Control mice underwent sham procedures that involved the same anesthesia administration but no irradiation.

Five mice per radiation dose and time point were sacrificed by intraperitoneal administration of pentobarbital (150 mg/kg) at 1, 2, 4, 8, 12, 16, 20, and 24 weeks after irradiation, and perfused with 4% paraformaldehyde buffer solution. Subsequent en-bloc excision of the irradiated spines was performed. Interlaminar horizontal sections of the resected specimens were histologically and immunohistochemically assessed along with the age-matched nonirradiated specimens (n = 5/time point) using a BZ-9000 microscope (Keyence, Osaka, Japan).

Additional 3 mice each were sacrificed at 1, 12, and 24 weeks after irradiation with 20 Gy, and perfused with 4% paraformaldehyde buffer solution. The excised spines were placed in fixative for electron microscopy analysis along with the age-matched nonirradiated specimens (n = 3/time point).

### Histologic assessment

**Fig 2:**
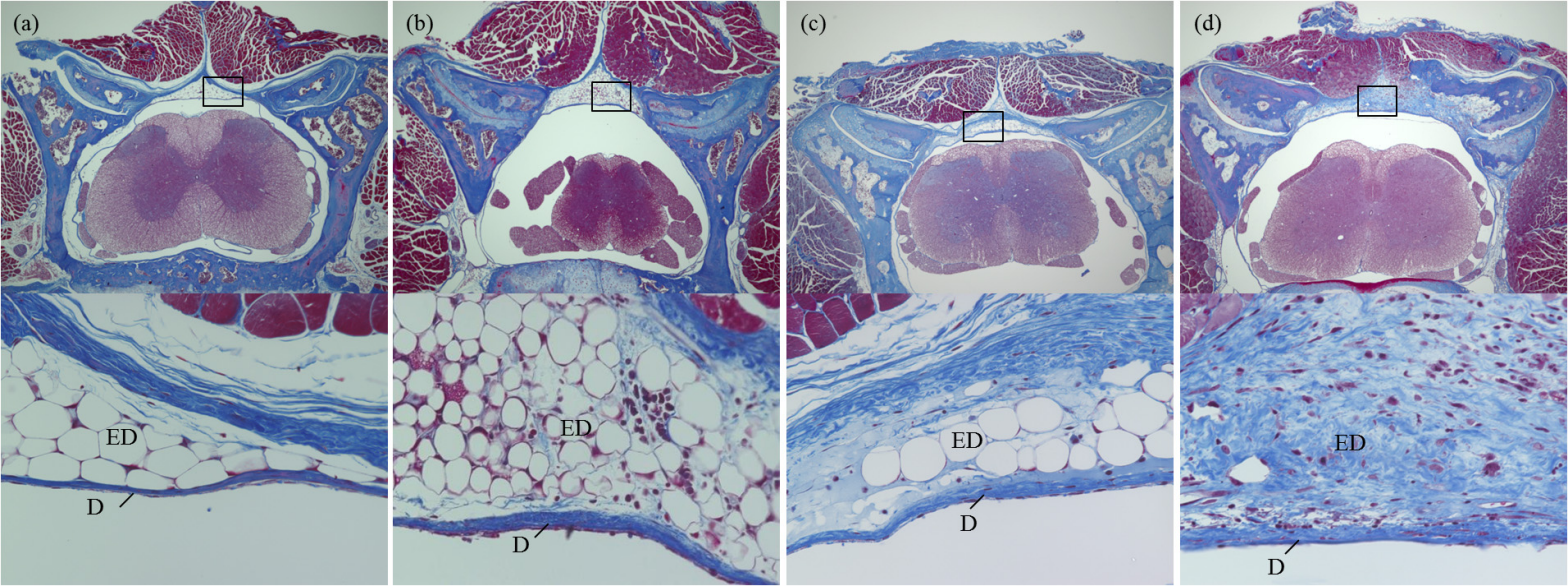
Representative histologic features of radiation-induced epidural fibrosis. (a) Grade 0, no fibrosis. (b) Grade 1, minimum fibrosis. (c) Grade 2, moderate fibrosis. (d) Grade 3, severe fibrosis. (Masson’s trichrome stain; above, magnification ×40; below, magnification ×400 objective). Abbreviations: D, dura mater; ED, epidural space.

For histologic assessment, the excised specimens were fixed in Tissue-Tek UFIX (Sakura Finetek Japan, Tokyo, Japan) for 3 days, and decalcified with formic acid for 1 week. Then, the specimens were embedded in paraffin and cut into 2-μm sections for Masson’s trichrome staining. Two blinded observers independently scored the epidural fibrosis in accordance with a modified postlaminectomy scar formation score from 0 (no fibrous tissue) to 3 (severe fibrosis) (Table 1) [16]. The mean value of 2 interlaminar sections from each specimen was considered as the mean fibrosis score. Fig 2 shows the representative histology of each grade.

**Table 1:** Criteria for grading epidural fibrosis.

Grade	Histologic features
0	No fibrous tissue
1	Only thin fibrous bands are observed in the epidural space
2	Continuous fibrosis is observed, but it affects less than two-thirds of the epidural space
3	Fibrosis is large, affecting more than two-thirds of the epidural space, and/or fibrosis extends to the nerve roots

### Immunohistochemical staining

**Fig 3:**
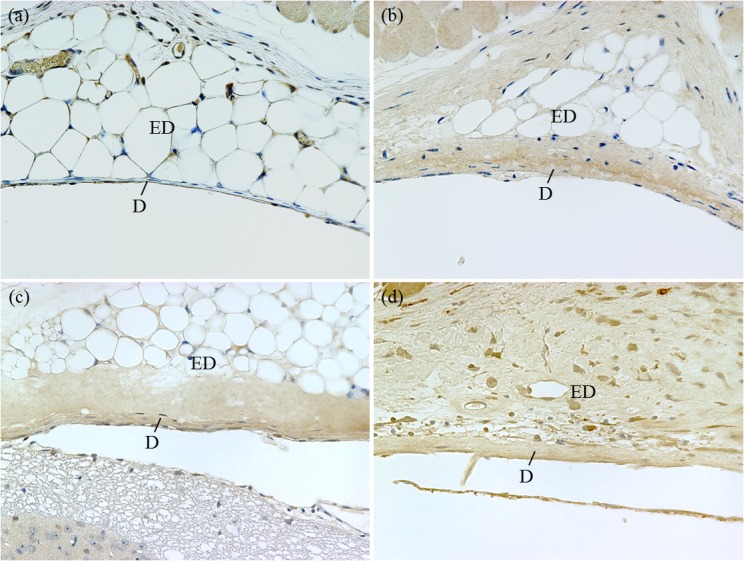
Immunohistochemical staining of TGF-β1. (a) Grade 0, no positive cells. (b) Grade 1, <30% of cells were positive. (c) Grade 2, 30%–59% of cells were positive. (d) Grade 3, ≥60% of cells were positive. (magnification ×400 objective). Abbreviations: D, dura mater; ED, epidural space.

Immunohistochemical staining for TGF-β1, which is thought to play a central role in fibrosis formation [17, 18], was performed to assess temporal expression in the dura mater. Sections (2-μm) of paraffin-embedded murine spine were cut, then deparaffinized in xylene and rehydrated via a graded ethanol series. Then, the sections were placed in an antigen-retrieval solution (L.A.B. Solution; PolySciences, Inc., Warrington, PA, USA) for 15 min at room temperature. After rinsing with deionized water, a peroxide-blocking reagent (0.3% hydrogen peroxide) was applied for 10 min at room temperature. After incubation in 5% bovine serum albumin in phosphate-buffered saline (PBS) for 10 min at room temperature, primary anti-TGF-β1 rabbit polyclonal antibody raised against the C-terminus of TGF-β1 of human origin (sc-146; Santa Cruz Biotechnology, Santa Cruz, CA, USA; 1:300 dilution in PBS; previous validation in [19]) was applied overnight in a humidified chamber at 4°C. Then, the slides were washed in PBS and incubated in the secondary antibody (biotin goat antirabbit Ig, 550358; BD Biosciences, San Jose, CA, USA) for 30 min at room temperature. The slides were then incubated with a streptavidin-horseradish peroxidase (550946; BD Biosciences) for 30 min at room temperature. After rinsing with PBS, peroxidase activity was visualized with 3, 3’-diaminobenzidine staining. Subsequently, the slides were washed 3 times for 10 min each in deionized water and counterstained with Mayer’s hematoxylin. As a negative control, primary antibodies were omitted. The rate of TGF-β1–positive cells was graded into 4 easily reproducible subcategories according to the frequency of positively stained cells as a percentage of total cell count: grade 0, no positive cells; grade 1, <30% of cells were positive; grade 2, 30%–59% of cells were positive; and grade 3, ≥60% of cells were positive. Fig 3 shows the representative histology of each grade. As with the histologic assessment, 2 blinded observers performed the scoring, and the mean value of 2 interlaminar sections for each specimen was taken as the mean positive TGF-β1 score.

### Assessment of microstructure using electron microscopy

For electron microscopy assessment, the excised specimens were fixed by immersion in 4% paraformaldehyde buffer solution for 4 h at 4°C, and subsequently decalcified in ethylenediaminetetraacetic acid for 3 to 4 days at 4°C. The tissue blocks were cut into 20-μm sections, fixed with 4% paraformaldehyde and 1% glutaraldehyde buffer solution for 30 min at 4°C, and posifixed with 1% OsO_4_ for 30 min at 4°C. Then, the specimens were stained with 2% uranium acetate for 30 min, dehydrated in a graded ethanol series, and embedded in an epoxy resin based on Glicidether (Selva Feinbiochemica, Heidelberg, Germany). Ultrathin sections (about 70 nm) were prepared using an ultramicrotome, stained with uranium acetate and lead citrate, and subjected to observation using a JEM-1210 electron microscope (JEOL, Tokyo, Japan).

### Statistical analyses

The extent of epidural fibrosis and the frequency of TGF-β1–positive cells in each group were compared using the 2-tailed Mann-Whitney *U* test. Statistical significance was set at a P value of < 0.05. SPSS version 19 (SPSS, Chicago, IL, USA) was used to perform the statistical analyses. All values in the figures indicate means and standard errors.

## Results

### Epidural fibrosis after irradiation

**Fig 4:**
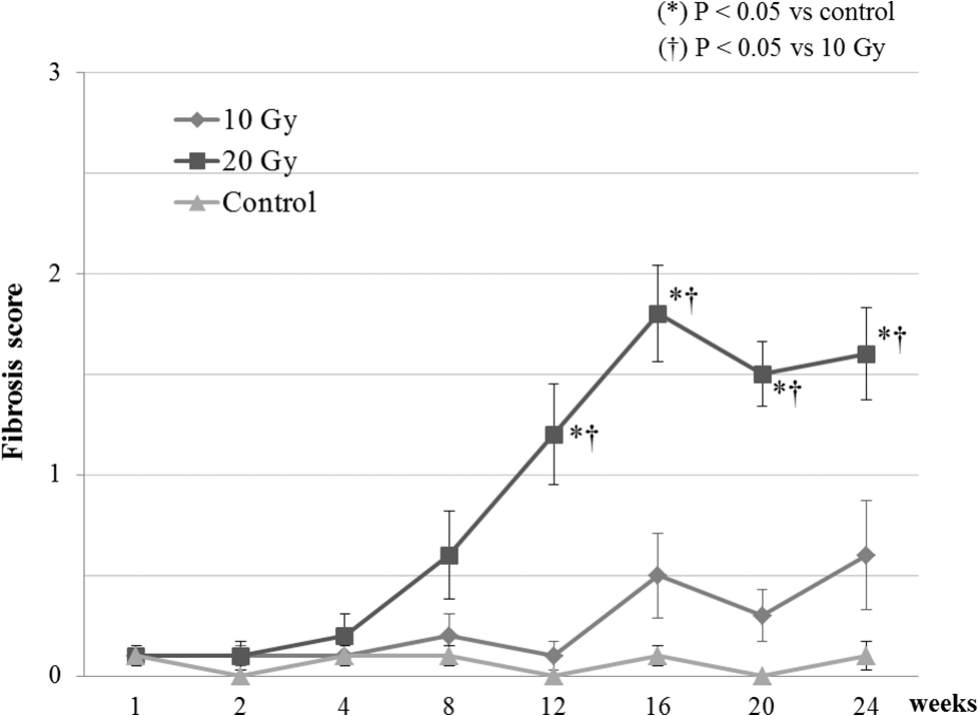
Epidural fibrosis scores of mice with 10 Gy or 20 Gy irradiation and without irradiation. The extent of epidural fibrosis was scored at each indicated time point (n = 5/time point).

In the nonirradiated mice, slight thin fibrous bands were observed in a few cases, and there was no certain occurrence of fibrosis. In the 10-Gy irradiated mice, continuous fibrosis was occasionally observed from 16 weeks postirradiation. In the 20-Gy irradiated mice, fibrous bands were observed from around 8 weeks postirradiation, and continuous fibrosis first occurred around 12 weeks; epidural fibrosis was observed in all cases from 16 weeks postirradiation. The extent of epidural fibrosis was significantly higher in the 20-Gy irradiated mice compared with that in the nonirradiated and 10-Gy irradiated mice from 12 weeks postirradiation (P < 0.05) (Fig 4).

### TGF-β1 levels

**Fig 5:**
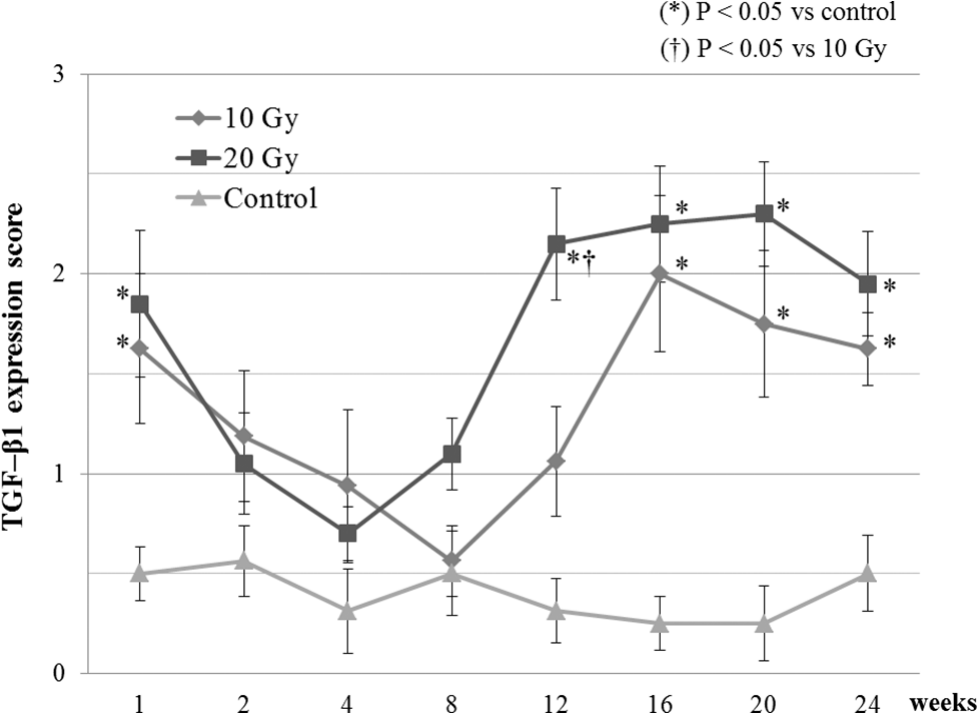
TGF-β1 expression in the dura mater of mice with 10 Gy or 20 Gy irradiation and without irradiation. The expression of TGF-β1 was scored at each indicated time point (n = 5/time point).

Compared with the nonirradiated mice, the 20-Gy irradiated mice showed significantly more overexpression of TGF-β1 at 1 week and ≥12 weeks postirradiation (P < 0.05); the 10-Gy irradiated mice showed significantly more overexpression of TGF-β1 at 1 week and ≥16 weeks postirradiation (P < 0.05). At 12 weeks postirradiation, the 20-Gy irradiated mice showed significantly more overexpression of TGF-β1 compared with the 10-Gy irradiated mice (Fig 5).

### Microstructure of dura mater and surrounding tissues

**Fig 6:**
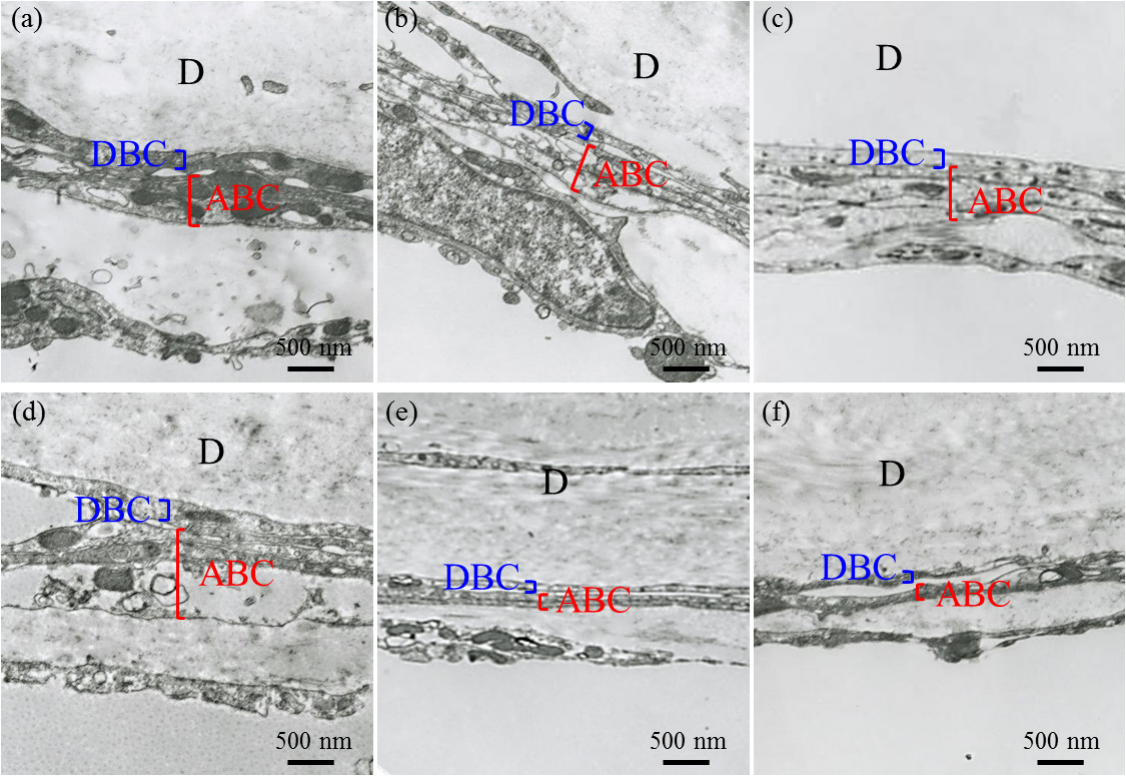
Microstructure of the dura mater and surrounding tissue. (Above) Nonirradiated mice at (a) 1 week, (b) 12 weeks, and (c) 24 weeks. (Below) 20-Gy irradiated mice at (d) 1 week, (e) 12 weeks, and (f) 24 weeks. (magnification ×10,000). Abbreviations: ABC, arachnoid barrier cell layer; D, dura mater; DBC, dural border cell layer.

There were no prominent changes in the microstructure of the dura mater between the 20-Gy irradiated and nonirradiated mice. On the other hand, the arachnoid barrier cell layer (ABC)—the outermost layer of the arachnoid mater—was enlarged at 1 week postirradiation and thinned at 12 and 24 weeks in the 20-Gy irradiated mice compared with in the nonirradiated mice (Fig 6). These findings were observed in all cases.

## Discussion

Radiation injury can be classified into 2 types [20–22]. Early injury occurs predominantly in the acute phase of the inflammatory response, and is characterized by angiogenic disorder and impaired tissue remodeling. On the other hand, late injury is typified by fibrosis and delayed wound healing caused by fibroblast dysfunction. Of these, radiation-induced fibrosis is regarded as a composite tissue reaction consisting of prominent extracellular matrix deposition and abnormal outgrowth of fibroblasts. Various growth factors and cytokines play important roles in this process; among them, TGF-β1 is thought to play a central role by acting as a master switch in inducing, forming, and maintaining fibrosis [17, 18]. Radiation-induced fibrosis has been reported in various organs, including the skin, lungs, kidneys, liver, and heart [13–15]. However, until now, there have been no reports focusing on the dura mater.

The dura mater is the outermost of the 3 meninges covering the spinal cord. Although there are slight differences in dural structure between humans and animals, it is composed mainly of fibroblasts with many extracellular collagen and elastic fibers, and adjacent fibroadipose tissue with a rich vasculature located in the epidural space [23, 24]. Epidural fibrosis was first described in 1948 as fibrous tissue deposits in the epidural space following posterior spinal surgery [25]. Since then, much research has been conducted, but almost all studies have investigated epidural fibrosis following spinal surgery [26, 27]. Epidural fibrosis is a contributing factor for failed back surgery syndrome [28], and is a strong risk factor for intraoperative dural injury due to severe adhesion [29]; there are currently no effective treatments for epidural fibrosis.

In the present study, we confirmed that epidural fibrosis was induced postirradiation in mice, and, despite the presence of individual differences, fibrosis was observed in all late stages after irradiation with 20 Gy. In addition, our results showed that epidural fibrosis was observed at a lower rate after irradiation with 10 Gy than after irradiation at 20 Gy. This finding is consistent with the findings of past reports in which a higher total dose was more likely to result in late-stage radiation injury [30, 31]. Moreover, overexpression of TGF-β1 was observed, suggesting a generation mechanism of epidural fibrosis that is similar to that observed in other tissue. Additionally, there were 2 peaks of overexpression: very early postirradiation and in the late stages after irradiation. This variation pattern of TGF-β1 expression is similar to a previous study that evaluated mice skin [32] and found that persistent overexpression of TGF-β1 in the late stages after irradiation is strongly involved in the occurrence of epidural fibrosis.

In clinical studies at our facility, occurrence of intraoperative dural injury was significantly higher at ≥12 months postirradiation than at <12 months [12]. In these cases, epidural fibrosis, as a late-stage radiation injury, could be a major cause of the subsequent dural injury. Therefore, because epidural fibrosis is more likely to occur in the late-stages after high-dose irradiation, surgery under such condition bears an increased risk of intraoperative dural injury associated with peridural adhesion. Moreover, when dural injury is observed during surgery, a primary suture is generally performed to prevent postoperative CSF leakage. This method is sufficient for treating most dural injuries. However, dural injury after irradiation easily develops into postoperative CSF leakage [33, 34]. In such cases, delayed wound healing as a late-stage radiation injury may occur in the dura mater due to radiation-induced cell dysfunction; however, no reports have verified this experimentally.

Another finding of the present study was post-irradiation thinning of the ABC. The ABC is located in the outermost layer of the arachnoid mater, and is formed of cells with tight junctions that are similar to endothelial cells [35]. The ABC has been reported to be strongly involved in meningeal permeability [36]. In addition, irradiation of vessels increases vascular permeability, and is associated with collapse of tight junctions in the vascular endothelial cell layer [37]. Therefore, it is anticipated that meningeal permeability would be increased in association with postirradiation ABC thinning by a similar mechanism. Clinically, postoperative CSF leakage is sometimes experienced despite the absence of dural injury [12]; the possibility that this would be caused by an increase in meningeal permeability due to radiation injury is fully conceivable. In the future, analysis of more samples and experimental verification of increased meningeal permeability associated with ABC thinning is necessary.

Because of the increase in the number of long-term survivors after radiotherapy for spinal metastases, most surgeons will encounter situations in which they will have to perform surgery on patients with a history of radiotherapy. Thus, full consideration should be given to late-stage radiation injury, including epidural fibrosis, and appropriate surgical strategies, meticulous surgical techniques, and measures for preventing serious complications should be employed. Recent studies have found that fibrosis is not untreatable, irreversible dead tissue, as had long been believed [13, 15]. Delanian et al. reported that application of combined pentoxifylline and tocopherol in patients with radiation-induced fibrosis elicited a 70% reduction in fibrotic volume, concomitant with a significant decrease in TGF-β1 expression [38]. Furthermore, this treatment proved efficacious in reducing fibrotic volume in a clinical randomized controlled trial [39]. In addition, Nishioka et al. reported that administration of a TGF-β-targeted drug before irradiation suppressed the occurrence of fibrosis [40]. Additional experimental research is in progress to evaluate the therapeutic effects of these drugs for epidural fibrosis.

The present study has an important limitation in that there are substantial differences in radiosensitivity between mice and humans [41]. This means that we could not estimate the dose volume and duration that would cause radiation-induced epidural fibrosis in humans. In addition, differences in cellular composition between mouse and human tissue could contribute to differences in molecular response [42]. Moreover, it is necessary to consider interhuman diversity. Strain differences in the radiation response of mice have been reported; for example, extensive fibrosis was observed in C57BL/6 mice, whereas it was almost absent in C3H mice [43]. It was also reported that inbred mice, which have been used extensively for modeling human diseases, are representative of a single individual and would not represent interhuman diversity [44]. In the present study, although closed-colony mice with genetic diversity were used rather than inbred mice, these too would not represent the full spectrum of interhuman diversity. Therefore, human subject research is indispensable; however, because acquiring dura mater and peridural tissue from the living body is difficult, postmortem research may be necessary.

In summary, this study confirms that epidural fibrosis is induced in the late stages after high-dose irradiation in mice, and that overexpression of TGF-β1 occurs in a manner similar to radiation-induced fibrosis in other tissue. Additionally, thinning of the ABC was observed in the late stages after irradiation. Thus, consideration should be given to the possibility that these phenomena can occur as radiation-induced injuries of the spine.

## Supporting Information

S1 ARRIVE Checklist (PDF)
